# Structure-Guided Identification of Phytochemical OCT2 Inhibitors and Their Functional Relevance to Cisplatin-Induced Cytotoxicity

**DOI:** 10.3390/pharmaceutics18040486

**Published:** 2026-04-15

**Authors:** Hyerim Song, Kyeong-Ryoon Lee, Hui Li, Mi-Kyung Lee, Yoon-Jee Chae

**Affiliations:** 1 College of Pharmacy, Woosuk University, Wanju 55338, Republic of Korea; 2 Laboratory Animal Resource Center, Korea Research Institute of Bioscience and Biotechnology, Cheongju 28116, Republic of Korea; 3 Department of Bioengineering, University of Science and Technology, Daejeon 34113, Republic of Korea; 4 Department of Pharmacology, College of Medicine, Konyang University, Daejeon 35365, Republic of Korea

**Keywords:** organic cation transporter 2, phytochemicals, structure–activity relationship, methoxylation, cisplatin-induced nephrotoxicity

## Abstract

**Background**: Organic cation transporter 2 (OCT2) mediates the renal uptake of cisplatin and is a principal contributor to its dose-limiting nephrotoxicity. Despite reports of OCT2 inhibition by various phytochemicals, the structure–activity relationships (SARs) governing inhibition and their functional implications remain poorly understood. **Methods**: We systematically evaluated OCT2 inhibitory activity across a structurally diverse library of 146 phytochemicals, including anthraquinones, flavanols, stilbenes, and isoflavones, using Madin–Darby canine kidney (MDCK) cells stably overexpressing OCT2. Structure–activity relationships were analyzed using non-parametric statistics and multivariate logistic regression, and functional relevance was assessed via cisplatin-induced cytotoxicity assays. **Results**: Inhibitory activity varied widely across the library, with potent inhibitors identified across multiple chemical scaffolds. Non-parametric statistical analyses revealed no significant differences in overall activity distributions among scaffold classes. Notably, chemical substituent patterns, rather than core scaffold identity, were the primary drivers of OCT2 inhibitory potency. Methoxylation was consistently associated with enhanced OCT2 inhibition, particularly within isoflavones, although its impact varied across structural scaffolds. The selected OCT2 inhibitors markedly reduced cisplatin-mediated cell death in OCT2-expressing cells but not in mock-transfected controls, confirming an OCT2-dependent mechanism of protection. **Conclusions**: This study establishes a structure-guided framework linking phytochemical OCT2 inhibition to nephroprotective potential and identifies methoxylation as a major determinant of OCT2-targeted intervention strategies.

## 1. Introduction

Cisplatin remains a cornerstone chemotherapeutic agent for a broad range of solid tumors; however, its clinical utility is constrained by dose-limiting toxicities—most prominently nephrotoxicity, which affects a substantial proportion of patients even under intensive prophylactic measures such as hydration and electrolyte supplementation [1]. At the cellular level, cisplatin-induced kidney injury preferentially targets the proximal tubular epithelial cells and triggers a complex network of stress responses, including DNA damage signaling, oxidative stress, mitochondrial dysfunction, inflammatory cascades, and regulated cell-death programs [2,3]. Because these downstream injury pathways are critically influenced by the degree of cisplatin accumulation within tubular cells, factors governing drug transport across cell membranes and intracellular drug exposure represent clinically important upstream determinants of toxicity.

Among the relevant kidney transport pathways, organic cation transporter 2 (OCT2; SLC22A2), a transporter located in the basolateral membrane of proximal tubule cells, has been repeatedly implicated as the primary entry route for cisplatin into these cells [4]. A seminal mechanistic study using human proximal tubule preparations identified OCT2 as a critical mediator of cisplatin-induced kidney injury and established a direct experimental link between OCT2-mediated drug transport and tubular cell damage [1]. Subsequent translational investigations further supported this model by demonstrating OCT2-dependent cisplatin handling and its relationship to kidney injury outcomes in patients [5,6]. Importantly, clinical and translational evidence indicates that functional variations in SLC22A2 are associated with inter-individual differences in nephrotoxicity risk, reinforcing the concept that OCT2-dependent uptake is a modifiable contributor to kidney injury [6]. Pharmacological inhibition of OCT2 has therefore emerged as a rational nephroprotective strategy, as reducing tubular drug uptake is expected to lower intracellular cisplatin levels and blunt downstream cytotoxicity cascades. A prototypical example is cimetidine, a well characterized OCT2 inhibitor that has been investigated as an adjunct therapy to reduce cisplatin nephrotoxicity both in vitro and in vivo [7].

Plant-derived bioactive compounds, particularly polyphenolic phytochemicals, represent a promising chemical space for identifying novel OCT2 inhibitors, as they offer broad scaffold structural diversity and dense, chemically modifiable substitution patterns within a shared aromatic framework. Polyphenols commonly display variable hydroxyl (–OH) and methoxy (–OCH3) groups that systematically modulate their hydrogen-bonding capacity, molecular planarity, and overall balance of water solubility and lipophilicity—properties known to influence interactions with broadly selective organic cation transporters (OCTs). Indeed, OCT-family transporters recognize a wide range of structurally distinct cationic molecules, and transporter-focused reviews have emphasized that the physicochemical features of a ligand, rather than its core structural class alone, often govern inhibition [8]. A structurally diverse phytochemical library therefore provides a practical basis for comparing inhibitor activity across distinct polyphenolic backbones and for deriving meaningful structure–activity relationships (SARs) within a controlled chemical space.

Consistent with this concept, prior studies have reported that selected flavonoids, a major class of plant phenols, can potently inhibit OCT2 and reduce cisplatin-induced cell death in OCT2-expressing cellular systems. Flavonoid-focused screening efforts identified multiple inhibitors active at low-micromolar concentrations and demonstrated that OCT2 inhibition translated into measurable protection against cisplatin-mediated cytotoxicity, with activity differences attributable to specific chemical substituents and substitution patterns [9,10]. While these findings provided initial proof-of-concept for phytochemical OCT2 inhibition, they were largely confined to a single structural family, limiting scaffold-level comparisons and broader generalization of SAR trends.

Furthermore, SAR insights from existing studies have often relied on limited analog series or position-specific observations, making it difficult to disentangle generalizable physicochemical drivers—such as the relative contributions of hydroxylation versus methoxylation—from effects specific to a given scaffold. Functional validation beyond uptake inhibition, particularly quantitative assessment of cisplatin cytotoxicity shifts in OCT2-expressing versus control cells, has typically been limited to small compound subsets. As a result, a unified framework linking scaffold diversity, quantitative inhibition potency, SARs, and nephroprotective functional outcomes remains lacking.

In the present study, we addressed these gaps by systematically screening a library of 146 phytochemicals encompassing four major polyphenolic scaffolds—anthraquinones, flavanols, stilbenes, and isoflavones—for their ability to inhibit OCT2-mediated transport. Compounds with potent inhibitory activity were further characterized through concentration–response analysis to determine half-maximal inhibitory concentration (IC_50_) values for OCT2 inhibition. Comprehensive SAR analyses were then performed using both univariate and multivariate statistical approaches, including structural-class–restricted analysis within isoflavones, the largest and most structurally diverse subclass, as well as matched-pair comparisons of structurally related compounds differing by a single chemical substitution, designed to isolate the contribution of specific structural features. Finally, we evaluated whether selected OCT2 inhibitors could reduce cisplatin-induced cytotoxicity by quantifying shifts in the concentration causing 50% cytotoxicity (CC_50_) in OCT2-expressing Madin–Darby canine kidney (MDCK-OCT2) versus mock-transfected control (MDCK-mock) cells, thereby directly linking transporter inhibition to nephroprotective potential. By integrating scaffold-level screening, quantitative potency data, and multilayered SAR analyses with functional cytotoxicity experiments, this study provides a structure-guided map of phytochemical OCT2 inhibition and establishes a direct link between transporter inhibition and reduced cisplatin-mediated cytotoxicity in an OCT2-dependent model. These findings identify structurally accessible natural-product frameworks and substitution patterns that can inform the rational design of nephroprotective OCT2 inhibitors and guide future evaluation of transporter-mediated drug interactions relevant to clinical cisplatin therapy.

## 2. Materials and Methods

### 2.1. Chemicals and Reagents

**Figure 1:**
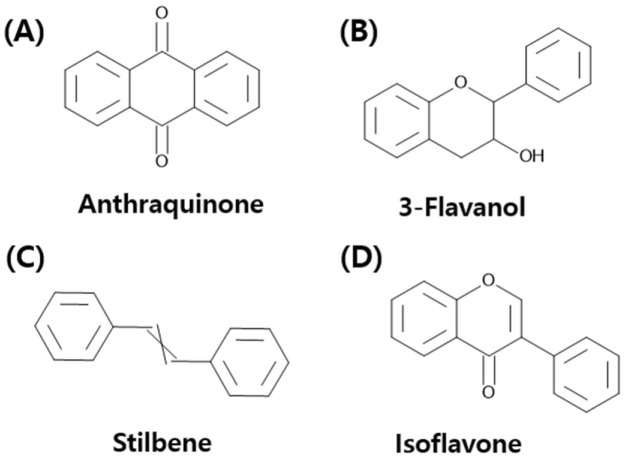
Chemical scaffolds of phytochemicals evaluated in this study. (**A**) Anthraquinone, (**B**) 3-flavanol, (**C**) stilbene, and (**D**) isoflavone core structures analyzed for OCT2 inhibitory activity.

A total of 146 compounds, comprising anthraquinones (*n* = 22), 3-flavanols (*n* = 24), stilbenes (*n* = 26), and isoflavones (*n* = 74), were provided by the Korea Chemical Bank (Daejeon, Republic of Korea) (Figure 1 and Supplementary Figure S1). Dulbecco’s Modified Eagle’s Medium (DMEM), phosphate-buffered saline (PBS), and Triton X-100 were purchased from Sigma-Aldrich (St. Louis, MO, USA). Rhodamine 123 was obtained from Invitrogen (Carlsbad, CA, USA), and cisplatin was purchased from Tokyo Chemical Industry (TCI, Tokyo, Japan). Fetal bovine serum (FBS), 4-(2-hydroxyethyl)-1-piperazineethanesulfonic acid (HEPES), non-essential amino acids (NEAA), Hank’s balanced salt solution (HBSS), and penicillin–streptomycin were supplied by Gibco (Grand Island, NY, USA). The tetrazolium dye 3-(4,5-dimethylthiazol-2-yl)-2,5-diphenyltetrazolium bromide (MTT), used in cell viability assays, was obtained from Alfa Aesar (Haverhill, MA, USA). All reagents were of analytical or reagent grade.

### 2.2. Cell Culture

MDCK-OCT2 and MDCK-mock cells [9,11,12] were maintained in high-glucose DMEM supplemented with 10% (*v*/*v*) heat-inactivated FBS, 100 U/mL penicillin, 0.1 mg/mL streptomycin, and 1% NEAA. Cells were cultured at 37 °C in a humidified incubator under a 5% CO_2_ atmosphere. To maintain optimal growth conditions, cells were routinely passaged upon reaching over 80% confluency.

### 2.3. Assessment of OCT2 Inhibitory Activity

The inhibitory effects of all 146 phytochemicals on OCT2-mediated transport were assessed with fluorescent substrate uptake assays using rhodamine 123 as the probe substrate. Cells were seeded in poly-D-lysine–coated 96-well plates at a density of 5 × 10^4^ cells/well and cultured for 24 h. Cells were washed twice with pre-warmed HBSS (pH 7.4) and were pre-incubated in HBSS at 37 °C for 20 min. Uptake was initiated by adding 0.5 µM rhodamine 123 in the presence or absence of phytochemicals at 10 µM. This concentration was selected to enable consistent comparison of inhibitory activity across a structurally diverse compound library for SAR analysis, rather than to reflect physiological exposure levels. Amodiaquine (20 µM) was used as a reference OCT2 inhibitor based on previous reports demonstrating its potent inhibition of OCT2-mediated transport, with IC_50_ values in the low micromolar range (1–11 µM) [9,13]. The selected concentration exceeds the reported IC_50_ values to ensure robust inhibition and to serve as a positive control for assay validation. The final concentration of dimethyl sulfoxide (DMSO) did not exceed 1% (*v*/*v*) in any experimental condition. After 10 min of incubation, the reaction was terminated by removing the incubation solution and washing immediately with ice-cold PBS. Cells were then lysed using 0.1% Triton X-100 in PBS [14,15]. Fluorescence intensity was measured at excitation/emission wavelengths of 485/535 nm using a Tecan Infinite M Plex microplate reader (Tecan, Männedorf, Switzerland) and normalized to total protein content, which was determined using a bicinchoninic acid (BCA) protein assay kit (iNtRON Biotechnology, Seongnam, Republic of Korea). Statistical significance was assessed using one-way analysis of variance (ANOVA) followed by Dunnett’s post hoc test for multiple comparisons against the vehicle control group.

For compounds showing greater than 50% inhibition of rhodamine 123 uptake at 10 μM, IC_50_ values were determined by exposing cells to 0.5 µM rhodamine 123 in the presence of varying concentrations of the test compound (0, 0.1, 0.4, 2, and 10 μM). Concentration–uptake activity data were fitted using a non-linear regression model in GraphPad Prism software (version 10.6.1; GraphPad Software Inc., La Jolla, CA, USA) according to the following equation:

$$Uptake\;activity\left(\%\;of\;control\right)=\frac{100}{1+{\left(\frac{C}{{IC}_{50}}\right)}^{n}}$$

where C is the test compound concentration and *n* is the Hill coefficient. Goodness-of-fit was evaluated using the coefficient of determination (R^2^), and all fitted curves showed satisfactory fit quality (R^2^ > 0.9).

### 2.4. SAR Analysis

All chemical structures were represented as canonical SMILES strings, and molecular descriptors and substructure features were calculated using RDKit-based algorithms implemented in Python (version 3.11). Physicochemical descriptors included molecular weight (MW), calculated octanol–water partition coefficient (cLogP), number of hydrogen bond donors (HBDs), and number of hydrogen bond acceptors (HBAs), parameters that capture the overall size, lipophilicity, and hydrogen-bonding capacity of each compound. In parallel, structure-based counts of key functional groups, including hydroxyl (–OH), methoxy (–OCH_3_), catechol, and glycoside-like motifs, were derived using SMARTS substructure pattern matching. These descriptors were selected to capture physicochemical and structural features relevant to the polyphenolic scaffolds examined and their potential interactions with OCT2.

OCT2 inhibitory activity was expressed as the percentage of rhodamine 123 uptake remaining relative to the vehicle control. For categorical analyses, compounds were classified as OCT2 inhibitors if remaining uptake activity fell below 50% (corresponding to ≥50% inhibition at the screening concentration). This classification threshold was applied consistently throughout subsequent analyses to distinguish compounds with functionally meaningful OCT2 inhibition from weak or negligible inhibitors. To assess the robustness of this classification, sensitivity analyses were performed using multiple alternative thresholds (30–70%).

To explore initial relationships between individual structural features and OCT2 inhibitory activity, univariate statistical analyses were first conducted across the full dataset. For continuous variables, including methoxy count, hydroxyl count, cLogP, MW, HBD, and HBA, Spearman’s rank correlation was applied to account for non-normal distributions. For binary structural features, such as the presence or absence of catechol, or glycoside-like motifs, differences in residual OCT2 activity were evaluated using the Mann–Whitney U test. In addition, differences in remaining OCT2 activity across scaffold (moiety) classes were assessed using the Kruskal–Wallis test. All univariate analyses were conducted as two-sided tests because no directional hypotheses were predefined. Univariate analyses were conducted in an exploratory manner to identify potential associations; therefore, multiple testing correction was not applied.

Multivariate logistic regression analyses were subsequently performed to identify independent predictors of strong OCT2 inhibition. OCT2 inhibitor status (residual activity of <50%) served as the binary outcome variable. The primary model included total methoxy count as the main explanatory variable, with total hydroxyl count, presence of catechol motifs, and presence of glycoside-like motifs included as covariates. Scaffold (moiety) class was incorporated as a set of indicator (dummy) variables to account for structural-class–dependent effects on inhibitory activity. All predictors were entered simultaneously without stepwise selection. Models were fitted using a logit link function and maximum likelihood estimation, and observations with missing descriptor values were excluded using listwise deletion. Odds ratios (ORs) and corresponding 95% confidence intervals (CIs) were derived by exponentiating the regression coefficients, and heteroscedasticity-robust standard errors were used to assess statistical significance.

Because isoflavones constituted the largest structural subset and displayed the widest range of OCT2 inhibitory activity in the primary screen, the same SAR analysis workflow was separately applied to the isoflavone subclass. All descriptors, statistical tests, and modeling approaches were identical to those used in the full-dataset analysis, except that scaffold indicator variables were omitted. To specifically evaluate the effect of O-methylation on OCT2 inhibitory activity, isoflavone pairs differing solely by hydroxy-to-methoxy substitution at equivalent positions were manually identified based on their chemical structures. For each matched hydroxy/methoxy isoflavone pair sharing the same core backbone and overall substitution pattern, residual OCT2 activity was directly compared. Differences in OCT2 inhibitory activity between hydroxy- and methoxy-substituted isoflavones were evaluated using a two-sided Wilcoxon signed-rank test.

All SAR-related statistical analyses were performed using Python-based workflows (version 3.11). Unless otherwise specified, all tests were two-sided, and statistical significance was defined as *p* < 0.05.

### 2.5. MTT Assay for Cisplatin-Induced Cytotoxicity

The ability of selected phytochemicals to reduce cisplatin-induced cytotoxicity was assessed using the MTT cell viability assay. MDCK-mock and MDCK-OCT2 cells were seeded in 96-well plates at a density of 2.4 × 10^4^ cells/well and incubated for 24 h, followed by treatment with cisplatin at a range of concentrations (0, 0.05, 0.5, 5, 20, and 50 µM) for 48 h. To evaluate protective effects, selected phytochemicals were co-administered with cisplatin at a non-toxic concentration of 10 µM for 48 h. After the treatment period, MTT solution was added to each well and incubated for 4 h. The resulting formazan crystals were dissolved in DMSO, and absorbance was measured at 540 nm using the Tecan Infinite M Plex microplate reader. Cell survival was expressed as a percentage of the untreated control.

The concentration of cisplatin producing 50% cell death (CC_50_) was determined to quantify shifts in cisplatin-induced cytotoxicity in the presence or absence of OCT2 inhibitors. Concentration–cell survival data were fitted using a non-linear regression model in GraphPad Prism software (version 10.6.1) according to the following equation:

$$Cell\;survival\;(\%\;of\;control)=\frac{100}{1+{\left(\frac{C}{{CC}_{50}}\right)}^{n}}$$

where C is the cisplatin concentration and *n* is the Hill coefficient.

## 3. Results

### 3.1. OCT2 Inhibitory Activity of Phytochemicals

**Figure 2:**
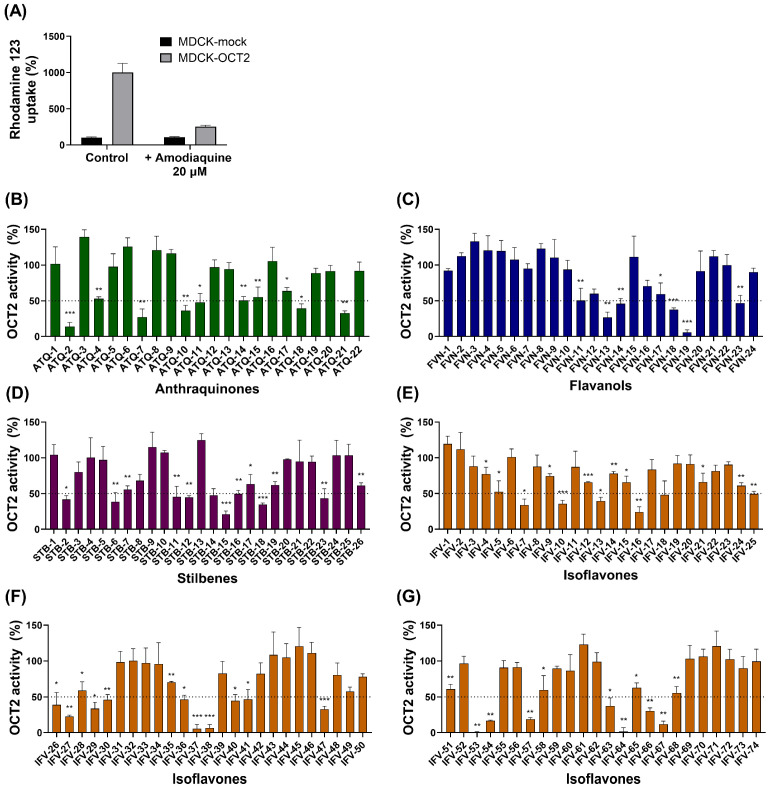
OCT2-mediated rhodamine 123 uptake and inhibition by phytochemicals. (**A**) Rhodamine 123 uptake in MDCK-mock and MDCK-OCT2 cells in the absence or presence of the reference OCT2 inhibitor amodiaquine (20 µM). (**B**–**D**) Effects of anthraquinones, flavanols, and stilbenes on OCT2 activity. (**E**–**G**) Effects of isoflavones on OCT2 activity. Cells were incubated with rhodamine 123 (0.5 µM) in the presence of phytochemicals (10 µM), and OCT2 activity is expressed as remaining uptake relative to control. The dotted line indicates the 50% threshold used to define OCT2 inhibition. Data are shown as mean ± SD (*n* = 3). Statistical significance versus control was determined using one-way ANOVA followed by Dunnett’s test (* *p* < 0.05, ** *p* < 0.01, *** *p* < 0.001).

A total of 146 phytochemicals were screened at 10 µM, and OCT2 activity was expressed as the percentage of rhodamine 123 uptake remaining relative to the vehicle control (Figure 2). Across the entire library, residual uptake activity ranged from 0.78% to 139.39%. Overall, 35 of the 146 phytochemicals (24.0%) reduced OCT2 activity by 50% or more. When compounds were stratified by structural class, the proportion exhibiting ≥50% OCT2 inhibition differed across classes: anthraquinones, 6/22 (27.3%); flavanols, 5/24 (20.8%); stilbenes, 3/26 (11.5%); and isoflavones, 21/74 (28.4%). A large portion of compounds showed weak or negligible inhibition, with 66/146 (45.2%) retaining 80–120% residual activity, whereas strong inhibition (<20% residual activity) was observed for only 9/146 compounds (6.2%).

The anthraquinone derivatives (*n* = 22) exhibited a broad range of OCT2 inhibitory effects, with residual activities spanning from 13.8% to 139.4%. Six compounds (27.3%) reduced OCT2 activity by at least 50%. Notably, closely related anthraquinones differing only in the position of dihydroxy substitution (e.g., ATQ-2, ATQ-3, ATQ-4, and ATQ-5) showed markedly different inhibitory profiles, highlighting the strong influence of substitution position within this scaffold.

Flavanols (*n* = 24) predominantly exhibited weak to moderate OCT2 inhibition, with residual activities ranging from 5.67% to 123.19%. Five compounds (20.8%) reduced OCT2 activity by ≥50%. Among them, silibinin showed near-complete inhibition and was the most potent flavanol identified. Overall, most flavanols displayed limited inhibitory activity.

Stilbenes (*n* = 26) showed generally weak OCT2 inhibition, with only 3 compounds (11.5%) reducing activity by ≥50%. Most stilbenes clustered within the weak or negligible inhibition range, indicating limited activity within this scaffold.

Isoflavones constituted the largest subgroup (*n* = 74) and displayed the widest distribution of OCT2 inhibitory activity, with residual activities ranging from 0.78% to 133.12%. Twenty-one of the 74 isoflavones (28.4%) reduced OCT2 activity by at least 50%. Several isoflavones produced near-complete inhibition, including IFV-53 (5,7-dimethoxy-6,8-dimethyl-4′-hydroxyisoflavone; 99.2% inhibition), IFV-64 [7-(2-methylbenzyloxy) isoflavone; 98.4% inhibition], and IFV-37 [2′-methoxyformonetin (7-hydroxy-2′,4′-methoxyisoflavone); 94.5% inhibition). In contrast, more than half of the isoflavones retained greater than 80% OCT2 activity, indicating weak or negligible inhibition.

### 3.2. OCT2 Inhibition Potency of Phytochemicals

**Figure 3:**
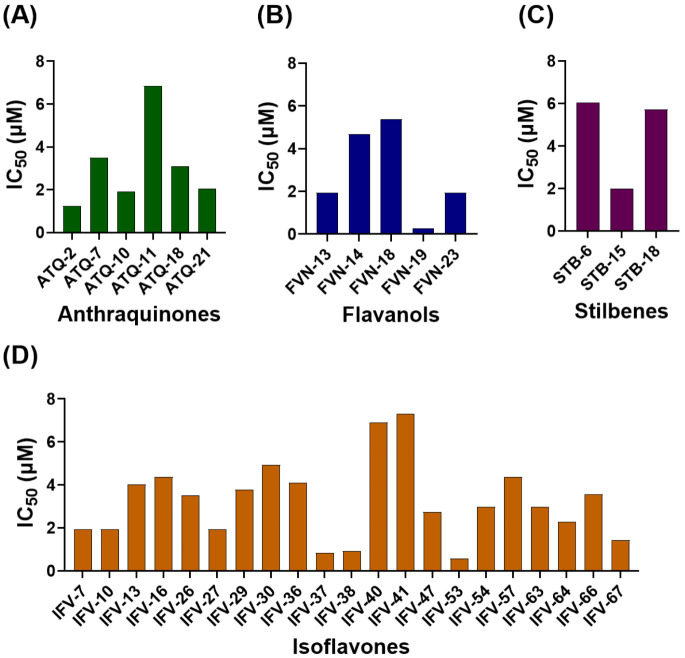
OCT2 inhibitory potency (IC_50_) of selected phytochemicals across different polyphenolic scaffolds. IC_50_ values were determined for phytochemicals that reduced OCT2 activity by ≥50% at 10 µM in the initial screening assay. OCT2 inhibition was quantified using a rhodamine 123 uptake assay in MDCK cells stably expressing human OCT2, and concentration–response curves were fitted by means of non-linear regression. (**A**) Anthraquinones, (**B**) flavanols, (**C**) stilbenes, and (**D**) isoflavones. Bars represent IC_50_ values (µM) derived from mean uptake data at each phytochemical concentration (*n* = 3).

Compounds that reduced OCT2 activity by at least 50% at 10 µM were subjected to concentration–response analysis to determine IC_50_ values, defined as the concentration required to inhibit OCT2 activity by 50%. A total of 35 compounds yielded quantifiable IC_50_ values, spanning a broad range from 0.27 µM to 7.32 µM (Figure 3, Table 1, and Supplementary Figure S2).

Several compounds exhibited sub-micromolar inhibitory potency (i.e., IC_50_ < 1 µM). The lowest IC_50_ was recorded for FVN-19 (silibinin; 0.27 µM). Other compounds active below 1 µM included IFV-53 (5,7-dimethoxy-6,8-dimethyl-4′-hydroxyisoflavone; 0.58 µM), IFV-37 [2′-methoxyformonetin (7-hydroxy-2′,4′-methoxyisoflavone); 0.85 µM], and IFV-38 (retusin 7-methyl ether; 8-hydroxy-7,4′-methoxyisoflavone; 0.92 µM).

The majority of compounds displayed IC_50_ values between 1 and 5 µM. Compounds with IC_50_ values above 5 µM included ATQ-11 [digitolutein (1-methoxy-2-hydroxy-3-methylanthraquinone); 6.86 µM], STB-6 [(E)-2-(methoxycarbonyl)stilbene; 6.04 µM], IFV-40 (6-isopropoxy-3′-methylisoflavone; 6.90 µM), and IFV-41 (7-isopropoxy-3′-methylisoflavone; 7.32 µM).

### 3.3. SAR Analysis

**Figure 4:**
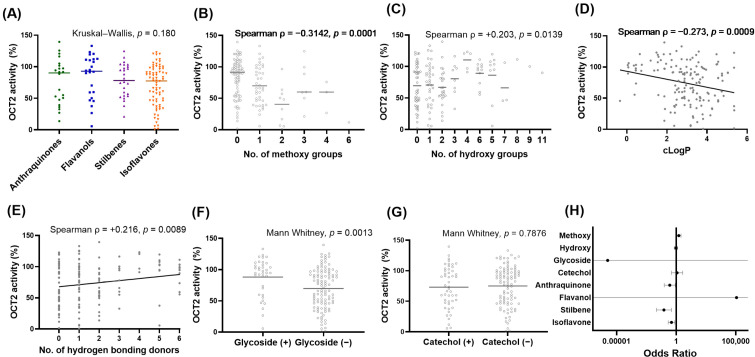
Structure–activity relationships of OCT2 inhibition across the full phytochemical library. (**A**) Comparison of OCT2 remaining activity among polyphenolic scaffolds (anthraquinones, flavanols, stilbenes, and isoflavones). (**B**–**E**) Correlations between OCT2 remaining activity and the number of methoxy groups (**B**), hydroxy groups (**C**), calculated lipophilicity (cLogP) (**D**), and hydrogen bond donors (**E**). (**F**,**G**) Comparison of OCT2 activity according to the presence or absence of glycoside (**F**) and catechol (**G**) motifs. (**H**) Odds ratios from multivariate logistic regression analysis identifying structural determinants of strong OCT2 inhibition across the full dataset.

OCT2 inhibitory activity was compared across the four structural scaffolds (anthraquinone, flavanol, isoflavone, and stilbene) using residual OCT2 activity as the primary measure (Figure 4). Non-parametric comparison across structural classes revealed no statistically significant difference in the overall distribution of residual OCT2 activity (Kruskal–Wallis test, H = 4.89, *p* = 0.180). Univariate Spearman’s rank correlation analysis was then performed to identify molecular features associated with OCT2 inhibition across the full dataset. A significant inverse correlation was observed between the total number of methoxy groups and residual OCT2 activity (ρ = −0.3142, *p* = 0.0001), indicating that compounds with methoxy substituents tended to show greater OCT2 inhibition. Similarly, cLogP showed a significant negative correlation with residual OCT2 activity (ρ = −0.273, *p* = 0.0009). In contrast, MW was not significantly correlated with OCT2 activity (ρ = −0.086, *p* = 0.300). HBD count and total hydroxyl group count showed weak but statistically significant positive correlations with residual OCT2 activity, meaning that compounds with more hydroxyl groups and HBDs tended toward weaker inhibition (HBD: ρ = +0.216, *p* = 0.0089; total hydroxyl group: ρ = +0.203, *p* = 0.0139).

To identify independent structural predictors of strong OCT2 inhibition, multivariate logistic regression was performed using a binary classification threshold of less than 50% residual activity (Figure 4). In the primary model, methoxy substitution was identified as a significant independent predictor of OCT2 inhibition (OR = 1.62, 95% CI: 1.06–2.49, *p* = 0.026), indicating that each additional methoxy group increased the odds of strong inhibition by approximately 62%. Sensitivity analyses using multiple inhibition thresholds (30–70% remaining activity) yielded consistent results, with methoxy substitution remaining positively associated with OCT2 inhibition across all thresholds (odds ratio range: 1.62–2.13, *p* < 0.05 for all). Structural class also contributed independently to the probability of strong inhibition. Compared with the reference class, isoflavones showed significantly lower odds of OCT2 inhibition (OR = 0.40, 95% CI: 0.21–0.76, *p* = 0.005). Similarly, stilbenes and anthraquinones showed lower odds (stilbene: OR = 0.09, *p* = 0.0015; anthraquinone: OR = 0.28, *p* = 0.021). Other structural features, including total hydroxyl count, were not significant independent predictors in the multivariate model (*p* > 0.6).

**Figure 5:**
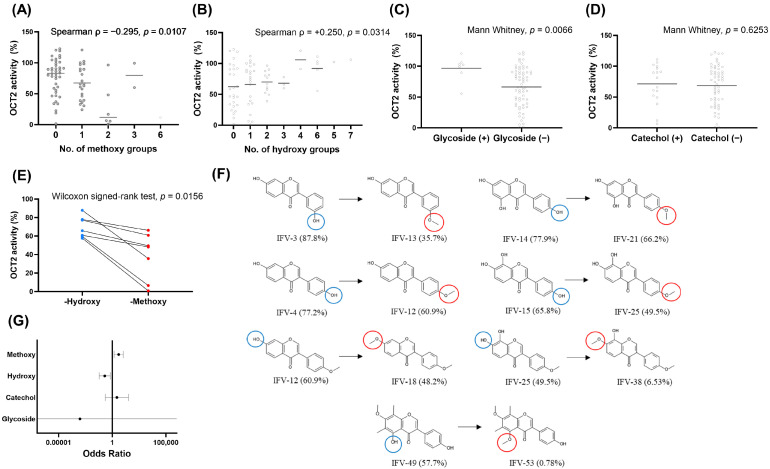
Structure–activity relationships of OCT2 inhibition within isoflavone derivatives. (**A**,**B**) Correlation between OCT2 remaining activity and the number of methoxy (**A**) or hydroxy (**B**) groups among isoflavones. (**C**,**D**) Comparison of OCT2 activity according to the presence of glycoside (**C**) or catechol (**D**) motifs in isoflavones. (**E**) Paired comparison of hydroxy- and methoxy-substituted isoflavones showing reduced OCT2 activity upon O-methylation. (**F**) Matched isoflavone pairs illustrating hydroxy-to-methoxy substitution and corresponding changes in OCT2 activity. Blue circles indicate hydroxy groups and red circles indicate methoxy groups. (**G**) Odds ratios from multivariate logistic regression analysis identifying substitution-level determinants of strong OCT2 inhibition within isoflavones.

Given that isoflavones represented the largest structural subset and displayed the widest range of OCT2 inhibitory activity, subsequent analyses were restricted to isoflavone derivatives (Figure 5). Within the isoflavone subset, total methoxy count remained significantly and inversely correlated with residual OCT2 activity (ρ = −0.295, *p* = 0.0107), confirming a consistent association between methoxylation and stronger inhibition within this class. Hydroxyl group count was positively correlated with residual activity, indicating weaker inhibition with increasing hydroxylation (ρ = +0.250, *p* = 0.0314). Multivariate logistic regression within the isoflavone subset confirmed the dominant role of methoxy substitution as an independent predictor of OCT2 inhibition. Within the isoflavone subset, methoxy count remained a strong independent predictor of OCT2 inhibition (OR = 3.93, 95% CI: 1.48–10.40, *p* = 0.00595), indicating a substantial increase in the likelihood of strong inhibition with increasing methoxylation. Total hydroxyl count was inversely associated with inhibition (OR = 0.20, 95% CI: 0.06–0.66, *p* = 0.00807). Glycoside substitution was significantly associated with OCT2 activity in the univariate analysis; however, this association was not retained in the multivariate logistic regression model. In contrast, the presence of a catechol moiety was not significantly associated with OCT2 inhibition in either the univariate or multivariate analyses.

To directly isolate the effect of O-methylation on OCT2 inhibitory activity, a pairwise analysis was performed using structurally matched hydroxy- and methoxy-substituted analogs (Figure 5). Across all seven compound pairs, the methoxy-substituted derivatives consistently showed lower residual OCT2 activity indicating greater inhibition than their hydroxy-substituted counterparts. A two-sided Wilcoxon signed-rank test confirmed a statistically significant difference between paired groups (W = 0.0, *p* = 0.0156), indicating that O-methylation was robustly associated with enhanced OCT2 inhibition within matched chemical backbones.

### 3.4. Attenuation of Cisplatin-Induced Cytotoxicity by Selected OCT2 Inhibitors

**Figure 6:**
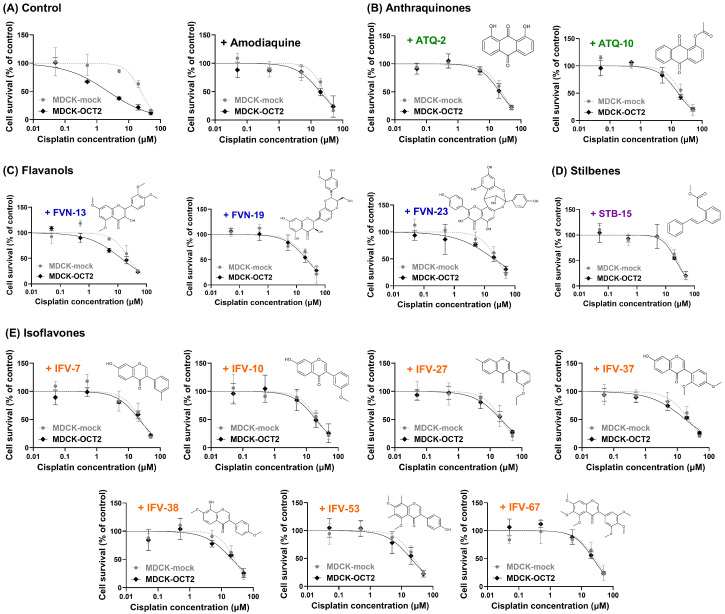
Attenuation of cisplatin-induced cytotoxicity by selected OCT2 inhibitors. Cisplatin concentration–cell survival curves were generated in MDCK-mock and MDCK-OCT2 cells using an MTT assay. (**A**) Control and reference OCT2 inhibitor amodiaquine. (**B**–**E**) Effects of selected anthraquinone (**B**), flavanol (**C**), stilbene (**D**) and isoflavone derivatives (**E**). Cells were treated with increasing concentrations of cisplatin in the absence or presence of test compounds (10 µM). Cell survival is expressed as a percentage of control. Data represent mean ± SD (*n* = 3).

The ability of selected compounds to reduce cisplatin-induced cytotoxicity was evaluated in MDCK-mock and MDCK-OCT2 cells using the MTT cell viability assay (Table 2 and Figure 6). Cell survival was assessed following 48 h exposure to increasing cisplatin concentrations in the absence or presence of test compounds co-incubated at 10 µM. In the absence of test compounds, MDCK-OCT2 cells were markedly more sensitive to cisplatin than MDCK-mock cells. The CC_50_ value of cisplatin causing 50% cell death was 24.1 µM in MDCK-mock cells, compared with 2.2 µM in MDCK-OCT2 cells, an approximately 11-fold difference confirming OCT2-dependent enhancement of cisplatin cytotoxicity. Co-administration of the reference OCT2 inhibitor amodiaquine produced a pronounced rightward shift in the cisplatin concentration–response curve in MDCK-OCT2 cells, with the CC_50_ increasing from 2.2 µM (control) to 19.3 µM, approaching the CC_50_ observed in MDCK-mock cells (25.7 µM). In contrast, amodiaquine produced minimal changes in cisplatin CC_50_ in MDCK-mock cells, indicating a selective protective effect in OCT2-expressing cells.

In MDCK-OCT2 cells, all tested scaffolds exhibited varying degrees of attenuation of cisplatin-induced cytotoxicity. Anthraquinone derivatives ATQ-2 and ATQ-10 markedly increased the cisplatin CC_50_ from 2.2 µM (control) to 20.8 and 16.6 µM, respectively. Flavanol-based compounds showed more heterogeneous effects: FVN-13 produced a moderate increase in CC_50_ (13.1 µM), whereas FVN-19 and FVN-23 increased CC_50_ to 22.6 and 20.3 µM, respectively. The stilbene derivative STB-15 also attenuated cisplatin cytotoxicity, increasing the CC_50_ to 22.8 µM in MDCK-OCT2 cells.

Isoflavone derivatives consistently shifted cisplatin concentration–response curves in MDCK-OCT2 cells, with CC_50_ values ranging from 18.3 µM (IFV-37) to 22.9 µM (IFV-67). Notably, highly methoxylated isoflavones such as IFV-53 and IFV-67 increased cisplatin CC_50_ values to 19.7 and 22.9 µM, respectively, indicating substantial attenuation of OCT2-mediated cisplatin cytotoxicity. In contrast, cisplatin CC_50_ values in MDCK-mock cells remained relatively constant across all treatment conditions, ranging from 21.6 to 25.9 µM, regardless of scaffold class or compound identity. This narrow variation indicates that the observed shifts in cisplatin cytotoxicity were largely restricted to OCT2-expressing cells, consistent with an OCT2-dependent mechanism of protection.

## 4. Discussion

In this study, we systematically evaluated OCT2 inhibition by phytochemicals spanning multiple polyphenolic structural classes and demonstrated that inhibitory activity is governed primarily by substituent patterns rather than core scaffold identity. Among the structural features examined, methoxylation emerged as a consistent determinant of OCT2 inhibition, particularly within isoflavones, although its magnitude varied across structural scaffolds. This scaffold-dependent variation likely reflects differences in structural diversity and substituent distribution across compound classes. Importantly, OCT2 inhibition by the selected phytochemicals translated into functional attenuation of cisplatin-induced cytotoxicity specifically in OCT2-expressing cells, supporting a direct mechanistic link between transporter inhibition and nephroprotective potential.

Prior studies have convincingly shown that selected polyphenols, particularly flavonoids, can inhibit OCT2 and reduce cisplatin-induced cytotoxicity in OCT2-expressing cellular systems [9,10]. However, earlier work was mostly confined to a limited number of compounds or a single chemical family, and SAR interpretations were often derived from qualitative comparisons or small sets of structurally related analogs. Consequently, it has remained unclear whether OCT2 inhibition by phytochemicals is primarily dictated by scaffold identity or by generalizable chemical features that apply to structurally distinct compound classes. The present study advances the field by adopting a scaffold-spanning, structure-guided framework coupled with functional validation. By evaluating anthraquinones, flavanols, stilbenes, and isoflavones within a unified experimental and analytical pipeline, we were able to directly compare inhibition patterns across structural classes while simultaneously investigating the effects of individual substituents. This integrative approach moves beyond earlier single-scaffold analyses and offers a more comprehensive picture of phytochemical OCT2 inhibition.

A key conceptual finding from this study is that scaffold identity alone does not dictate the overall inhibitory profile of polyphenolic compounds. Although different structural classes displayed broad and partially overlapping distributions of residual OCT2 activity, no statistically significant differences were observed in the overall activity distributions across classes. This observation is consistent with the well-established broad ligand selectivity of OCT-family transporters. These transporters recognize and transport a wide range of structurally diverse molecules, which makes them sensitive to the physicochemical properties of a ligand rather than to its specific structural class [8]. Nevertheless, multivariate analyses revealed that scaffold identity influenced the probability of compounds exceeding the strong inhibition threshold. Thus, although structural class does not strongly affect the average level of inhibition, it does modulate the likelihood that a given compound will qualify as a potent inhibitor. Importantly, this scaffold-dependent tendency appeared to reflect the enrichment of favorable substituents within particular classes, rather than an intrinsic property of the scaffold itself or bias introduced by unequal class sizes. This distinction reconciles the apparent discrepancy between distribution-level and threshold-based analyses and highlights the importance of analytical perspective when interpreting SAR data for transporter inhibition.

Within this framework, methoxylation emerged as a reproducible determinant of OCT2 inhibition. Earlier flavonoid-focused reports have suggested that methylated derivatives may exhibit stronger transporter inhibition than their hydroxylated counterparts; however, such observations have generally been based on limited datasets. In the present study, methoxy substitution was consistently associated with stronger OCT2 inhibition across multiple complementary analytical approaches, including univariate correlations, multivariate regression modeling, scaffold-restricted analyses, and matched-pair comparisons. The effect was most pronounced within the isoflavone subclass, which constituted the largest and most structurally diverse group in the library. This finding suggests that methoxylation represents an important substituent-level feature contributing to OCT2 inhibition within this chemotype, although its effect may not be equally pronounced across all structural classes. The convergence of results across independent analytical layers strengthens the robustness and generalizability of this conclusion. From a physicochemical perspective, methoxy substitution increases lipophilicity and reduces hydrogen-bond donor capacity, which may favor interactions with hydrophobic regions within the OCT2 binding environment. These changes may facilitate stronger transporter–ligand interactions and contribute to the increased inhibitory activity observed for methoxylated compounds.

In contrast to methoxylation, other functional groups showed more limited or context-dependent associations with OCT2 inhibition. Glycoside substitution showed a significant association with OCT2 activity in univariate analysis but did not retain significance in multivariate models, indicating that its apparent effect may largely reflect correlations with other co-occurring structural features. Similarly, the presence of a catechol group was not significantly associated with OCT2 inhibition in either univariate or multivariate analyses, suggesting that this structural feature does not play a dominant role in determining inhibitory activity within the present library. Hydroxylation exhibited a more nuanced pattern. Across the broader dataset, higher hydroxyl group counts and greater HBD capacity were associated with higher residual OCT2 activity, indicating a general tendency toward weaker inhibition with increasing hydroxylation. However, this relationship was not consistently retained as an independent predictor in multivariate analyses of the full dataset, likely because of confounding from structural class heterogeneity and co-occurring substitution patterns. Notably, when the analysis was restricted to isoflavones—a chemically more homogeneous subset—hydroxyl count emerged as a significant negative determinant of strong OCT2 inhibition. This finding suggests that the influence of hydroxylation becomes more apparent within a controlled structural framework and highlights the value of scaffold-restricted analyses for extracting clearer SARs.

cLogP showed a significant association with OCT2 inhibition in univariate analysis, suggesting an apparent contribution of lipophilicity. However, cLogP was not included in the multivariate logistic regression models, which were intentionally designed to focus on explicit structural features rather than composite physicochemical descriptors. This modeling strategy reflects the strong collinearity between cLogP and substitution-level variables such as methoxylation. Accordingly, the univariate lipophilicity signal likely represents a proxy for the underlying substitution patterns rather than an independent contribution of bulk lipophilicity itself. This interpretation highlights the importance of multivariate modeling for distinguishing true structural drivers of transporter inhibition from correlated physicochemical descriptors.

Beyond uptake inhibition, a major advance of the present work is the integration of functional validation using cisplatin-induced cytotoxicity as a clinically relevant endpoint. While earlier studies have often inferred nephroprotective potential based solely on transporter inhibition [16], the present study directly demonstrates that selected phytochemical OCT2 inhibitors attenuate cisplatin-induced cytotoxicity in an OCT2-dependent manner. The observation that protection was evident in MDCK-OCT2 cells but not in MDCK-mock cells argues against nonspecific cytoprotective mechanisms, such as antioxidant effects, and instead supports reduced OCT2-mediated cisplatin uptake as the dominant contributor. This functional link places the identified phytochemicals in a translationally meaningful context and strengthens the rationale for OCT2-targeted nephroprotective strategies.

In the present study, SAR analyses were based primarily on residual activity obtained from the initial screening assays, rather than on IC_50_ values or cisplatin cytotoxicity endpoints. This approach reflects the structure of the dataset and the study design. IC_50_ determination was performed only for compounds that already exhibited strong inhibition at the screening concentration, resulting in a relatively narrow potency range among the tested compounds. Under such conditions, small experimental variability may exert a disproportionately large influence on apparent SAR trends when IC_50_ values are modeled quantitatively. Similarly, cisplatin cytotoxicity assays were conducted on a selected subset of potent inhibitors to establish functional relevance, rather than to support quantitative SAR modeling. Accordingly, remaining OCT2 activity in the primary screen provided a more robust and uniform metric for capturing substitution-level effects across the full chemical space, while avoiding overinterpretation of minor potency differences among already potent inhibitors.

Several limitations should be considered when interpreting these findings. First, OCT2 inhibition was assessed using rhodamine 123 as a probe substrate. Although rhodamine 123 is widely used and has been characterized as a high-affinity OCT2 substrate, its intracellular accumulation can be influenced by mitochondrial membrane potential and efflux transporters such as p-glycoprotein (p-gp) [17,18,19]. To minimize these confounding effects, uptake measurements were conducted over a short incubation period (10 min), a condition selected to primarily capture the initial transporter-mediated influx rather than secondary intracellular processes. Experiments were performed using cells selectively overexpressing OCT2, allowing OCT2-mediated uptake to be evaluated under conditions in which contributions from other transport pathways, including p-gp–mediated efflux, were minimized. Second, scaffold-level comparisons should be interpreted with caution, as the compound library was not evenly distributed across structural classes. Isoflavones constituted approximately half of the dataset, whereas the other scaffold classes were represented by smaller numbers of compounds. This imbalance inherently limits statistical power for detecting scaffold-level differences and may obscure more subtle class-specific tendencies. However, this limitation does not undermine the substitution-level conclusions, which are supported by multiple complementary analyses. Indeed, the scaffold imbalance motivated the scaffold-restricted analyses within isoflavones, where sufficient chemical diversity enabled more reliable SAR extraction. Third, functional protection against cisplatin toxicity was assessed using cell viability rather than by direct measurement of intracellular cisplatin accumulation. Although the observed attenuation of cisplatin-induced cytotoxicity strongly suggests reduced intracellular drug accumulation via OCT2 inhibition, direct quantification of intracellular cisplatin levels (e.g., using ICP-MS or atomic absorption spectroscopy) was not performed in this study. Such measurements would provide more definitive mechanistic confirmation and represent an important direction for future investigation. In addition, given the structural diversity and pleiotropic nature of phytochemicals, potential interactions with other transporters, such as OCT1 and multidrug and toxic compound extrusion 1/2-K (MATE 1/2-K), cannot be excluded. While the use of OCT2-overexpressing and mock control cells allows isolation of OCT2-dependent effects, additional studies evaluating transporter selectivity would be required to fully characterize off-target interactions and their potential contribution to cisplatin disposition.

Taken together, the present findings provide a refined framework for understanding phytochemical OCT2 inhibition that integrates scaffold diversity, substitution-level SAR, and functional relevance. By demonstrating that OCT2 inhibition is driven primarily by substitution-level features—rather than scaffold identity per se—and by identifying O-methylation as a major determinant of OCT2 inhibition, particularly within isoflavones, we clarify previously ambiguous SAR trends reported in the literature. Importantly, the linkage between OCT2 inhibition and selective attenuation of cisplatin-induced cytotoxicity in OCT2-expressing cells underscores the mechanistic and translational relevance of these structural insights.

## 5. Conclusions

This study provides a systematic and structure-guided evaluation of phytochemical OCT2 inhibition and its functional relevance to cisplatin-induced cytotoxicity. By integrating scaffold-spanning screening, multilayered SAR analyses, and OCT2-dependent cytotoxicity assays, we demonstrated that OCT2 inhibitory activity is governed primarily by chemical substituent patterns rather than structural class identity, with O-methylation emerging as a major determinant of OCT2 inhibition, particularly within isoflavone derivatives, although its impact may depend on the structural scaffold. Importantly, OCT2 inhibition by selected phytochemicals substantially attenuated cisplatin-induced cytotoxicity in OCT2-expressing cells, supporting a mechanistically grounded nephroprotective strategy. Collectively, these findings advance the current understanding of polyphenol–transporter interactions and provide a rational framework for the future development and evaluation of OCT2-targeted nephroprotective agents.

## Figures and Tables

**Table 1:** IC_50_ values of selected phytochemicals for OCT2 activity in MDCK-OCT2 cells.

ID	Compound Name	IC_50_ (μM)
ATQ-2	1,8-Dihydroxyanthraquinone	1.24
ATQ-7	1,2-Dihydroxy-3-methylanthraquinone	3.49
ATQ-10	1-Acetoxyanthraquinone	1.91
ATQ-11	Digitolutein (1-methoxy-2-hydroxy-3-methylanthraquinone)	6.86
ATQ-18	1-Hydroxy-2-(methoxymethoxy)-3-methylanthraquinone	3.09
ATQ-21	Aurantioobtusin (1,3,7-trihydroxy-2,8-dimethoxy-6-methylanthraquinone)	2.04
FVN-13	3-Hydroxy-5,7,3′,4′-tetramethoxyflavanol	1.94
FVN-14	Catechin 7-O-apiofuranoside	4.68
FVN-18	Isosilybinin	5.38
FVN-19	Silibinin	0.27
FVN-23	Phenyl-substituted theaflavin analog	1.94
STB-6	(E)-2-(Methoxycarbonyl)-stilbene	6.04
STB-15	Phoyunbene A [(E)-3,3′-dihydroxy-2′,4′,5-trimethoxystilbene]	1.98
STB-18	(E)-3,5-Diacetoxy-4′-methoxystilbene	5.73
IFV-7	7-Hydroxy-3′-methylisoflavone	1.93
IFV-10	7-Hydroxy-3′-methoxyisoflavone	1.95
IFV-13	7-Hydroxy-2′-methoxyisoflavone	4.03
IFV-16	6-Methyl-4′-methoxyisoflavone	4.37
IFV-26	7,8-Dihydroxy-3′-methoxyisoflavone	3.52
IFV-27	7-Methyl-3′-ethoxyisoflavone	1.95
IFV-29	Ipriflavone (7-isopropoxyisoflavone)	3.79
IFV-30	2,2′-Methyl-7-methoxyisoflavone	4.93
IFV-36	7-Hydroxy-4′-acetoxyisoflavone	4.1
IFV-37	2′-Methoxyformonetin (7-hydroxy-2′,4′-methoxyisoflavone)	0.85
IFV-38	Retusin 7-methyl ether (8-hydroxy-7,4′-methoxyisoflavone)	0.92
IFV-40	6-Isopropoxy-3′-methylisoflavone	6.9
IFV-41	7-Isopropoxy-3′-methylisoflavone	7.32
IFV-47	7-Isopropoxy-4′-methoxyisoflavone	2.74
IFV-53	5,7-Dimethoxy-6,8-dimethyl-4′-hydroxyisoflavone	0.58
IFV-54	5-Hydroxy-6,8-dimethyl-7,4′-dimethoxyisoflavone	2.98
IFV-57	Daidzein diacetate (7,4′-diacetoxyisoflavone)	4.38
IFV-63	7-Benzyloxy-4′-methylisoflavone	2.99
IFV-64	7-(2-Methylbenzyloxy)-isoflavone	2.3
IFV-66	7-Benzyloxy-2-methyl-2′-methoxyisoflavone	3.57
IFV-67	Irigenin trimethyl ether (5,6,7,3′,4′,5′-hexamethoxyisoflavone)	1.44

**Table 2:** Effects of selected OCT2 inhibitors on cisplatin-induced cytotoxicity in MDCK-mock and MDCK-OCT2 cells.

ID	Name	MDCK-Mock	MDCK-OCT2
-	Control	24.1	2.2
-	Amodiaquine	25.7	19.3
ATQ-2	1,8-Dihydroxyanthraquinone	24.4	20.8
ATQ-10	1-Acetoxyanthraquinone	21.6	16.6
FVN-13	3-Hydroxy-5,7,3′,4′-tetramethoxyflavanol	24.5	13.1
FVN-19	Silibinin	24.4	22.6
FVN-23	Phenyl-substituted theaflavin analog	24.2	20.3
STB-15	Phoyunbene A [(E)-3,3′-dihydroxy-2′,4′,5-trimethoxystilbene]	25.4	22.8
IFV-7	7-Hydroxy-3′-methylisoflavone	23.7	21.8
IFV-10	7-Hydroxy-3′-methoxyisoflavone	22.2	19.9
IFV-27	7-Methyl-3′-ethoxyisoflavone	22.4	21.9
IFV-37	2′-Methoxyformonetin (7-hydroxy-2′,4′-methoxyisoflavone)	22.7	18.3
IFV-38	Retusin 7-methyl Ether (8-hydroxy-7,4′-methoxyisoflavone)	25.3	21.6
IFV-53	5,7-Dimethoxy-6,8-dimethyl-4′-hydroxyisoflavone	24.9	19.7
IFV-67	Irigenin trimethyl ether (5,6,7,3′,4′,5′-hexamethoxyisoflavone)	25.9	22.9

## Data Availability

The original contributions presented in this study are included in the article/supplementary material. Further inquiries can be directed to the corresponding author.

## Supplementary Materials

The following supporting information can be downloaded at https://www.mdpi.com/article/10.3390/pharmaceutics18040486/s1. Figure S1: Chemical structures of phytochemicals used in this study; Figure S2: Concentration-OCT2 activity curves to determine IC_50_ values of selected phytochemicals in MDCK-OCT2 cells.
