# Supplementary material for: Structure-Guided Identification of Phytochemical OCT2 Inhibitors and Their Functional Relevance to Cisplatin-Induced Cytotoxicity
Source: Pharmaceutics. 2026 Apr 15;18(4):486. doi: 10.3390/pharmaceutics18040486 (PMC13119655; doi:10.3390/pharmaceutics18040486)

Supplementary Materials

Supplementary Figure S1. Chemical structures of phytochemicals used in this study. (A) Anthraquinones (B) 3-Flavanols (C) Stilbenes (D) Isoflavones

(A) Anthraquinone (ATQ) derivatives

ATQ-1	ATQ-2	ATQ-3	ATQ-4
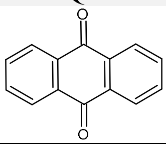	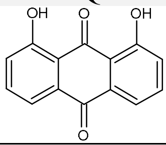	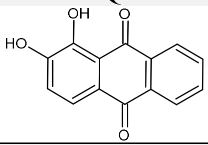	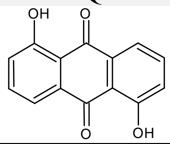
ATQ-5	ATQ-6	ATQ-7	ATQ-8
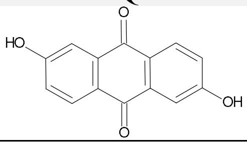	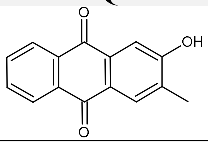	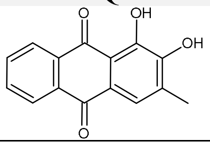	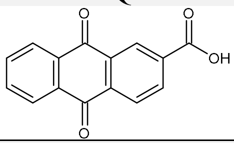
ATQ-9	ATQ-10	ATQ-11	ATQ-12
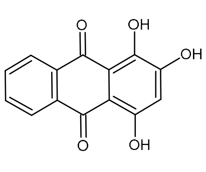	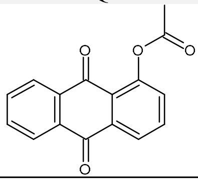	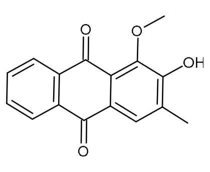	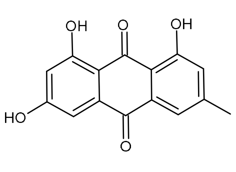
ATQ-13	ATQ-14	ATQ-15	ATQ-16
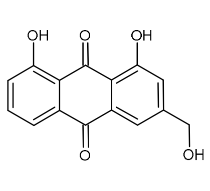	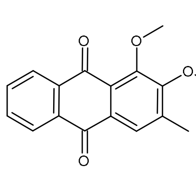	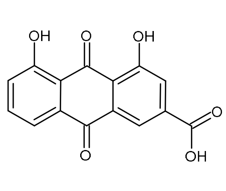	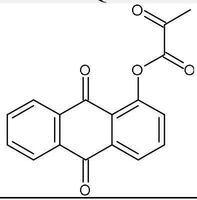
ATQ-17	ATQ-18	ATQ-19	ATQ-20
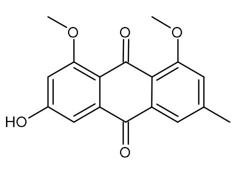	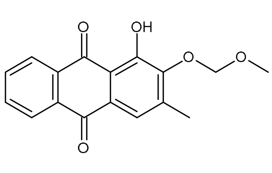	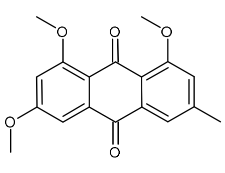	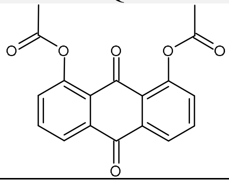
ATQ-21	ATQ-22		
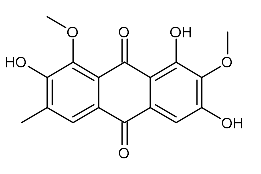	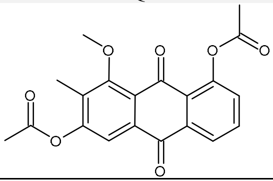		

(B) 3-Flavanol (FVN) derivatives

FVN-1	FVN-2	FVN-3	FVN-4
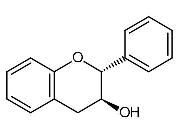	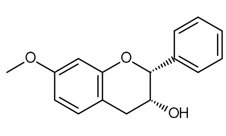	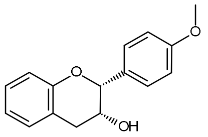	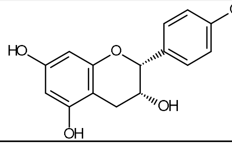
FVN-5	FVN-6	FVN-7	FVN-8
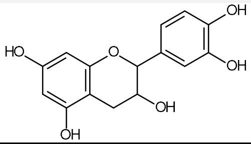	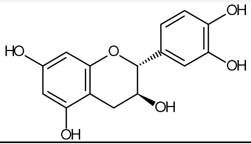	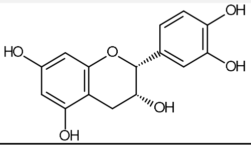	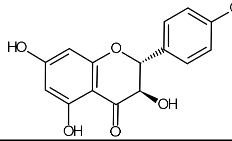
FVN-9	FVN-10	FVN-11	FVN-12
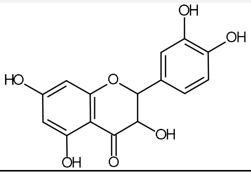	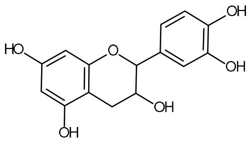	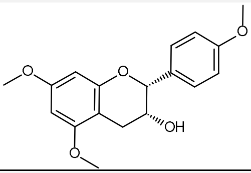	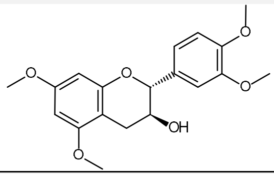
FVN-13	FVN-14	FVN-15	FVN-16
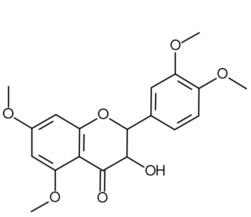	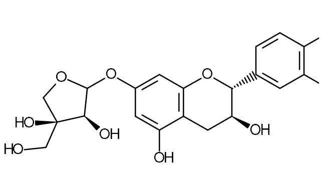	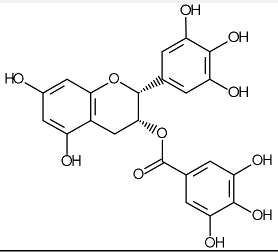	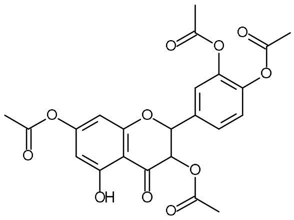
FVN-17	FVN-18	FVN-19	FVN-20
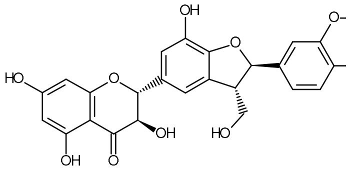	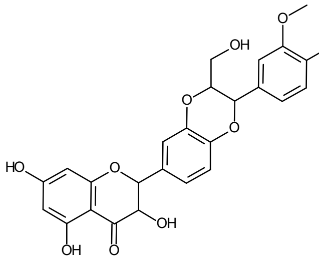	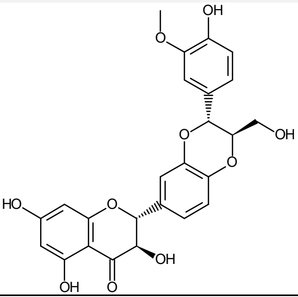	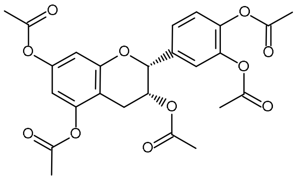
FVN-21	FVN-22	FVN-23	FVN-24
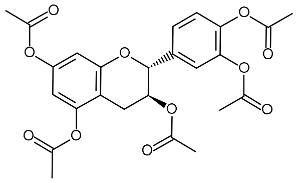	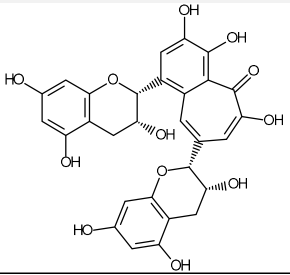	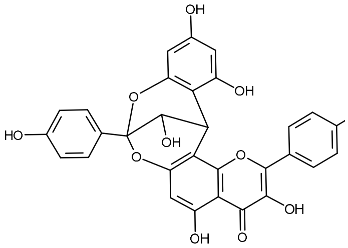	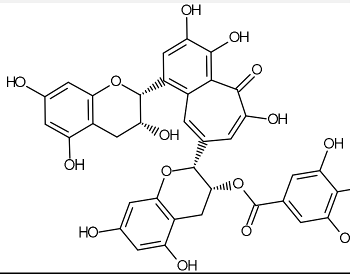

(C) Stilbene (STB) derivatives

STB-1	STB-2	STB-3	STB-4
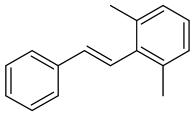	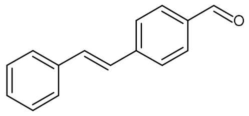	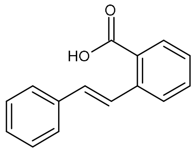	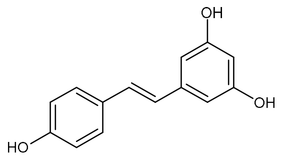
STB-5	STB-6	STB-7	STB-8
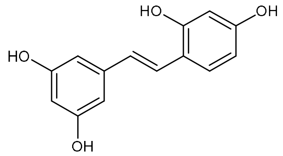	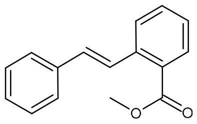	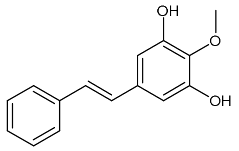	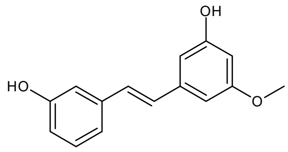
STB-9	STB-10	STB-11	STB-12
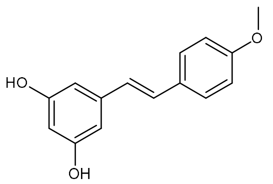	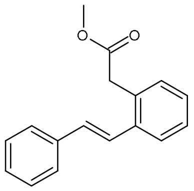	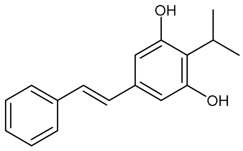	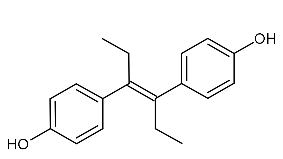
STB-13	STB-14	STB-15	STB-16
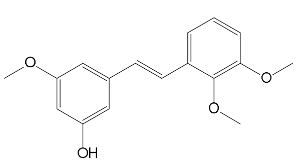	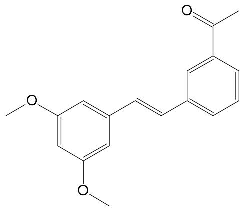	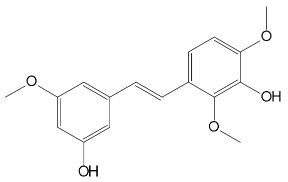	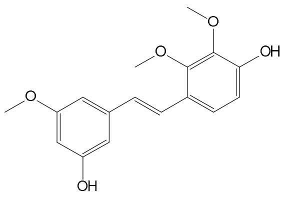
STB-17	STB-18	STB-19	STB-20
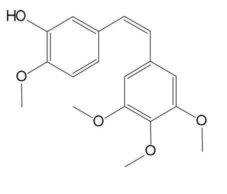	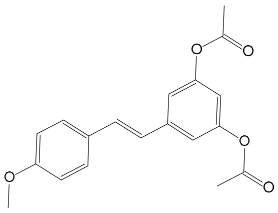	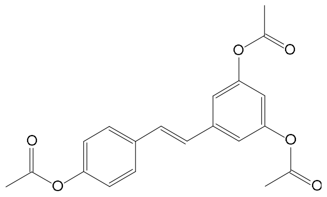	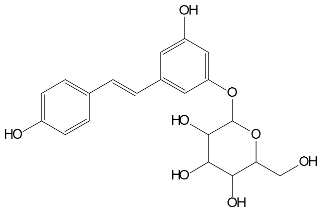
STB-21	STB-22	STB-23	STB-24
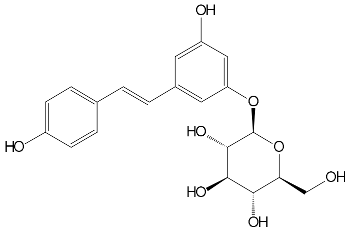	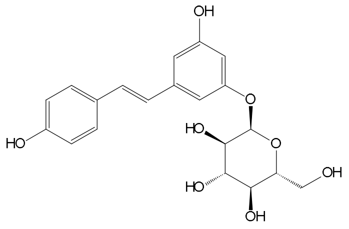	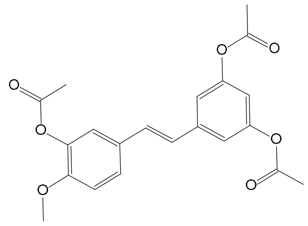	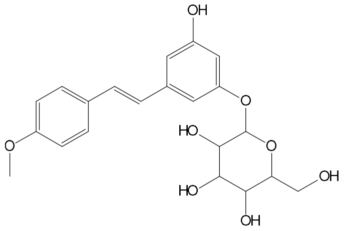
STB-25	STB-26		
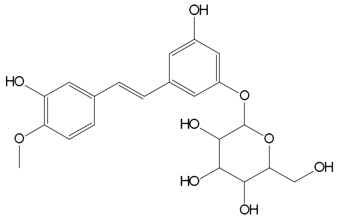	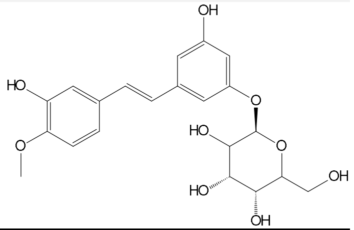		

(D) Isoflavone (IFV) derivatives

IFV-1	IFV-2	IFV-3	IFV-4
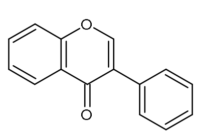	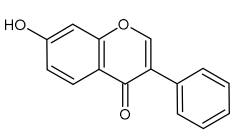	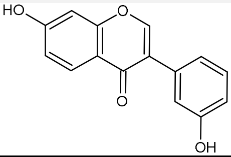	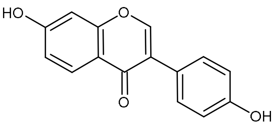
IFV-5	IFV-6	IFV-7	IFV-8
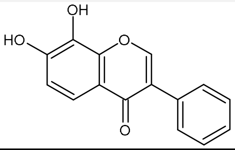	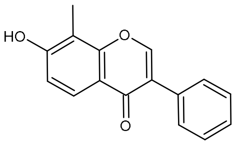	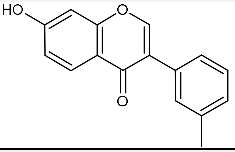	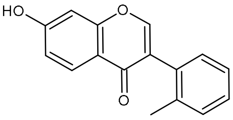
IFV-9	IFV-10	IFV-11	IFV-12
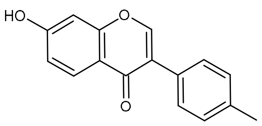	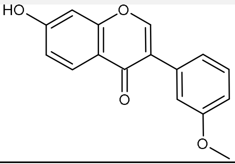	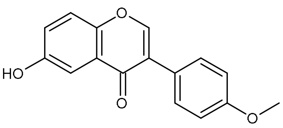	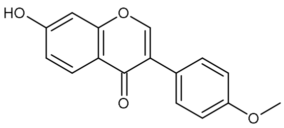
IFV-13	IFV-14	IFV-15	IFV-16
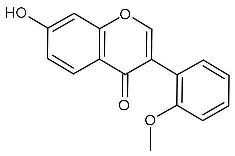	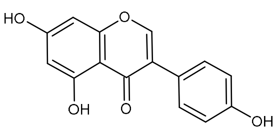	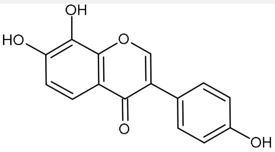	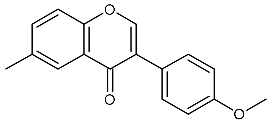
IFV-17	IFV-18	IFV-19	IFV-20
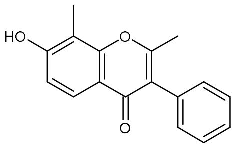	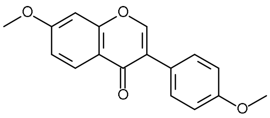	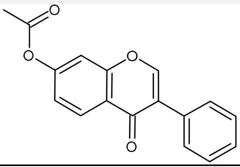	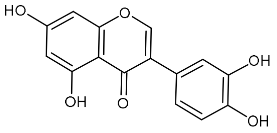
IFV-21	IFV-22	IFV-23	IFV-24
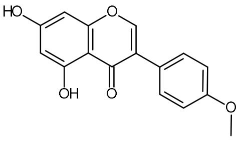	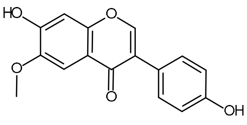	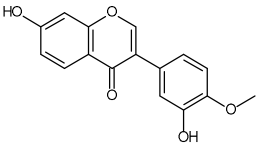	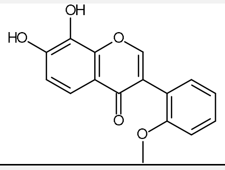
IFV-25	IFV-26	IFV-27	IFV-28
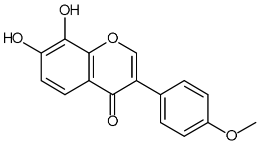	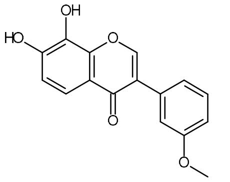	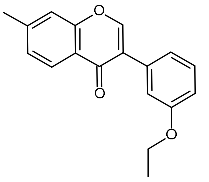	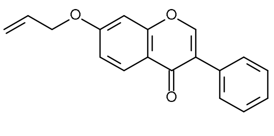
IFV-29	IFV-30	IFV-31	IFV-32
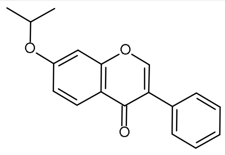	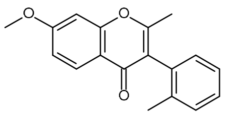	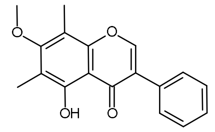	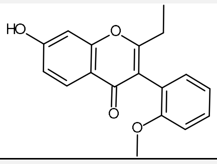
IFV-33	IFV-34	IFV-35	IFV-36
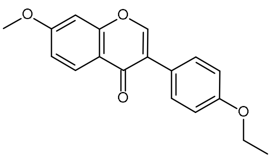	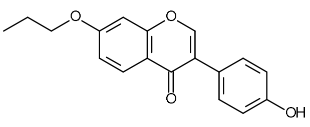	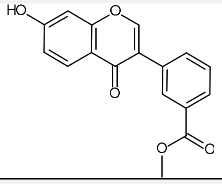	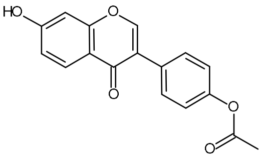
IFV-37	IFV-38	IFV-39	IFV-40
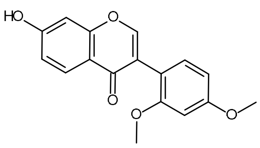	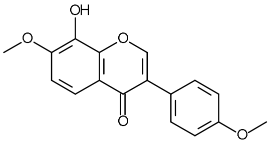	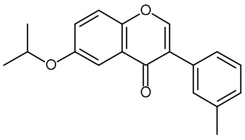	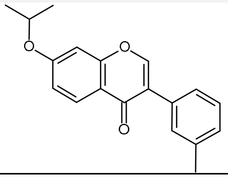

IFV-41	IFV-42	IFV-43	IFV-44
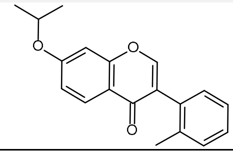	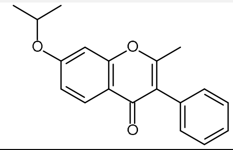	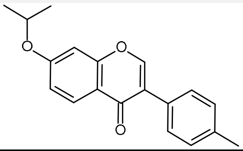	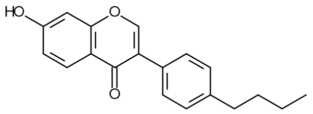
IFV-45	IFV-46	IFV-47	IFV-48
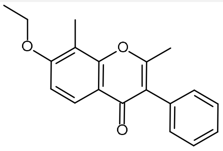	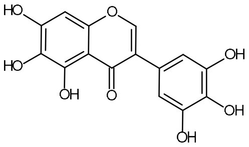	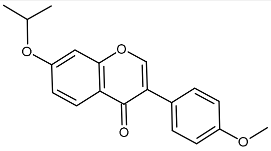	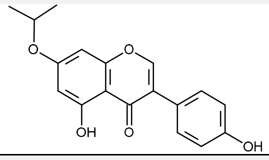
IFV-49	IFV-50	IFV-51	IFV-52
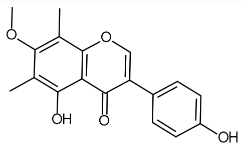	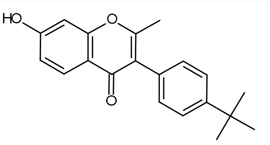	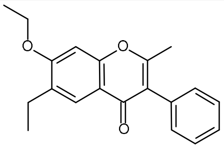	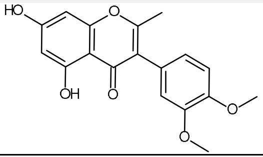
IFV-53	IFV-54	IFV-55	IFV-56
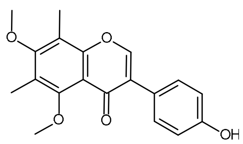	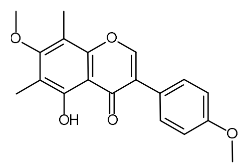	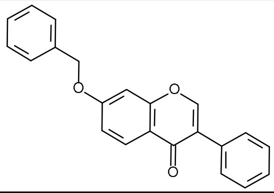	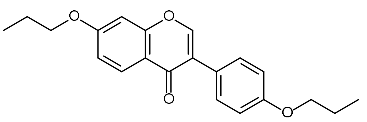
IFV-57	IFV-58	IFV-59	IFV-60
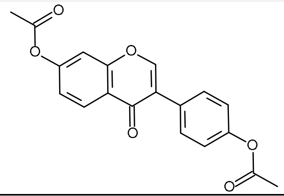	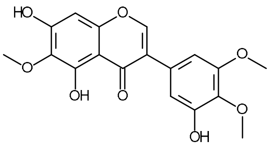	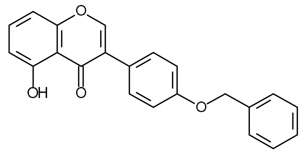	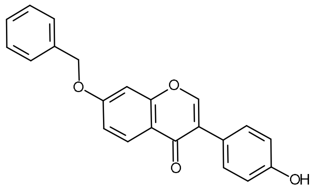
IFV-61	IFV-62	IFV-63	IFV-64
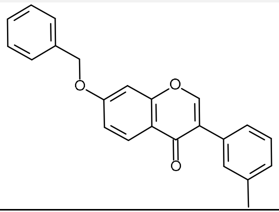	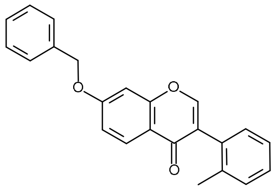	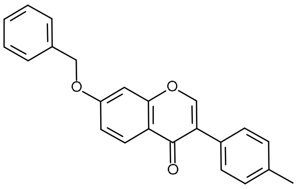	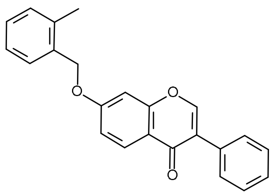
IFV-65	IFV-66	IFV-67	IFV-68
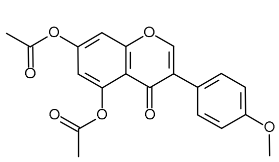	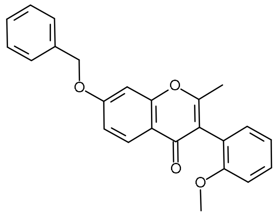	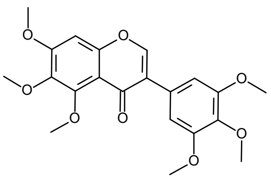	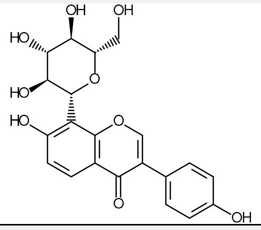
IFV-69	IFV-70	IFV-71	IFV-72
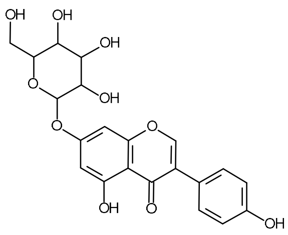	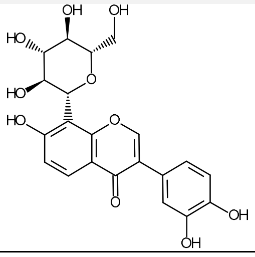	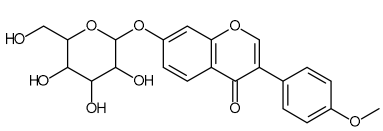	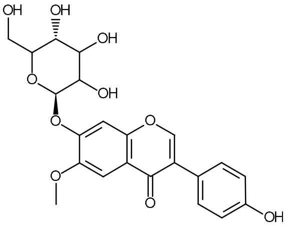

IFV-73	IFV-74
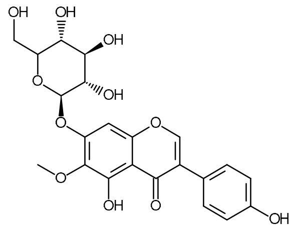	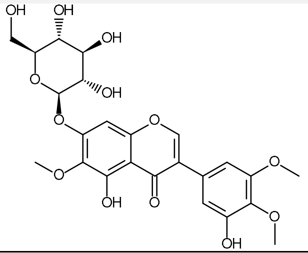

Supplementary Figure S2. Concentration-OCT2 activity curves to determine IC_{50} values of selected phytochemicals in MDCK-OCT2 cells. (A) Anthraquinones (B) 3-Flavanols (C) Stilbenes (D) Isoflavones

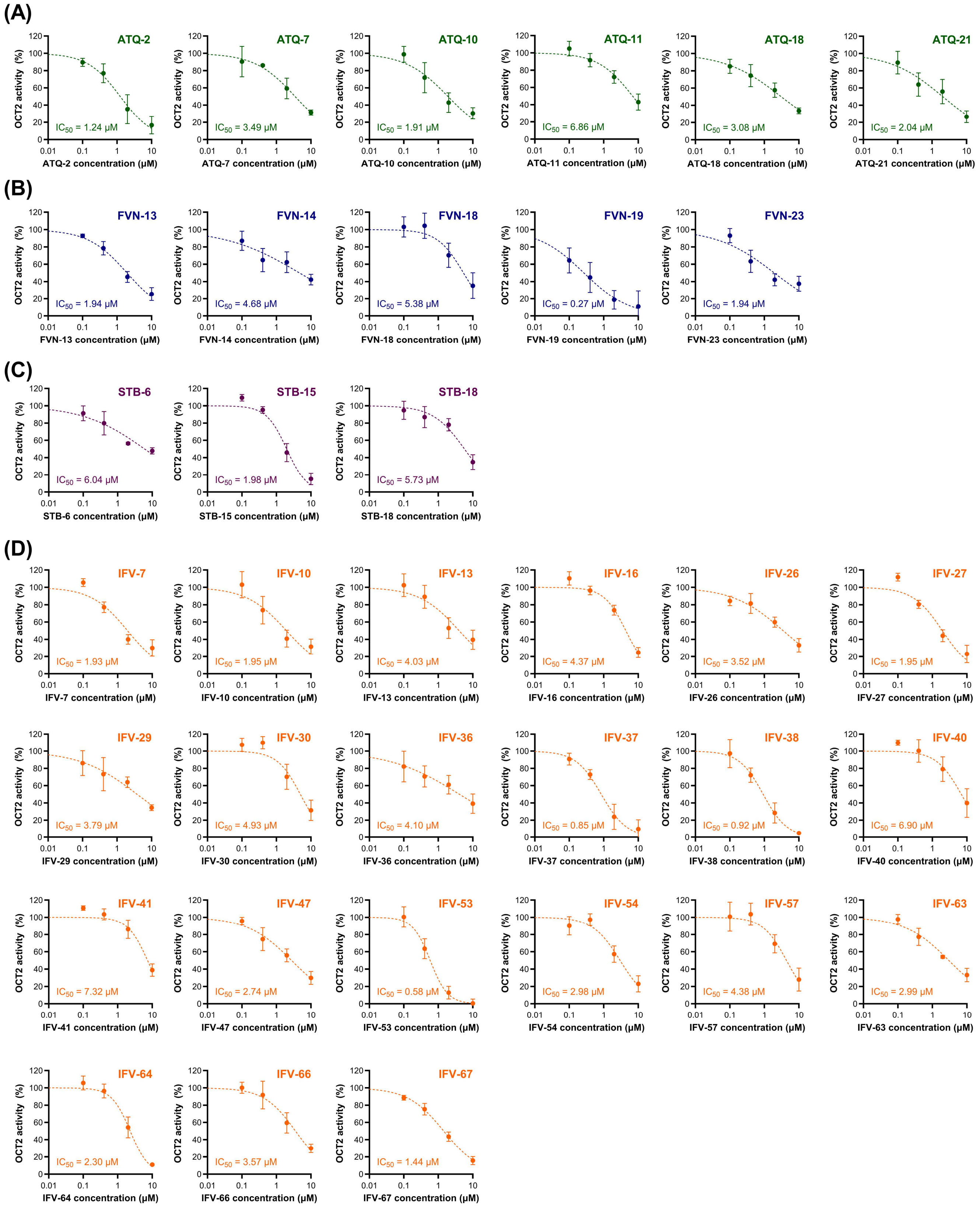

Supplement: Supplementary file 1 [file pharmaceutics-18-00486-s001.zip › pharmaceutics-4238030-supplementary.pdf]
